# Cutaneous Leishmaniasis Acquired in Jura, France

**DOI:** 10.3201/eid1801.110408

**Published:** 2012-01

**Authors:** William R. Faber, Rick Hoekzema, Aldert Bart, Jim E. Zeegelaar, Henry J.C. de Vries

**Affiliations:** Academic Medical Centre, Amsterdam, the Netherlands

**Keywords:** vector-borne infections, cutaneous leishmaniasis, sandflies, France, the Netherlands, climate change

**To the Editor:** Cutaneous leishmaniasis is well established in the Mediterranean basin. However, the disease is spreading and new foci have been reported (*1*–*3*). Because of climate change, it is feasible that vector-borne diseases such as cutaneous leishmaniasis may spread northward into Europe (*4*). We report a patient who acquired cutaneous leishmaniasis while on holiday in Jura, France.

A previously healthy 49-year-old white man from the Netherlands traveled to France in August 2007. During August 2–17, he stayed at a camp site in Clairvaux-les-Lacs in a forested area near a lake. He made regular trips by foot in the surrounding area. Three months later, he noticed a swelling on his nose. In February 2008, he consulted a dermatologist who treated him twice with cryotherapy under a diagnosis of actinic keratosis, after which the lesion nearly disappeared.

Three months later, the patient again consulted the dermatologist when the lesion recurred. A biopsy result from the lesion was interpreted as an acute ulcerative inflammation without further specification. Treatment was continued with imiquimod 5% cream followed by erythromycin 2% cream with clobetasol 0.05% ointment. Because of a lack of improvement, this treatment regimen was alternated with tacrolimus 0.1% ointment until May 2008.

In November 2008, he consulted another dermatologist, who obtained a biopsy specimen in which a large number of intracellular microorganisms compatible with leishmaniasis were observed in histiocytes. The patient was then referred to the Department of Dermatology at the Academic Medical Centre in Amsterdam. On examination, we found a plaque with a crusting surface and an erythematous border on the bridge of the nose (Figure, panel A). Regional lymph nodes were not palpable.

**Figure:**
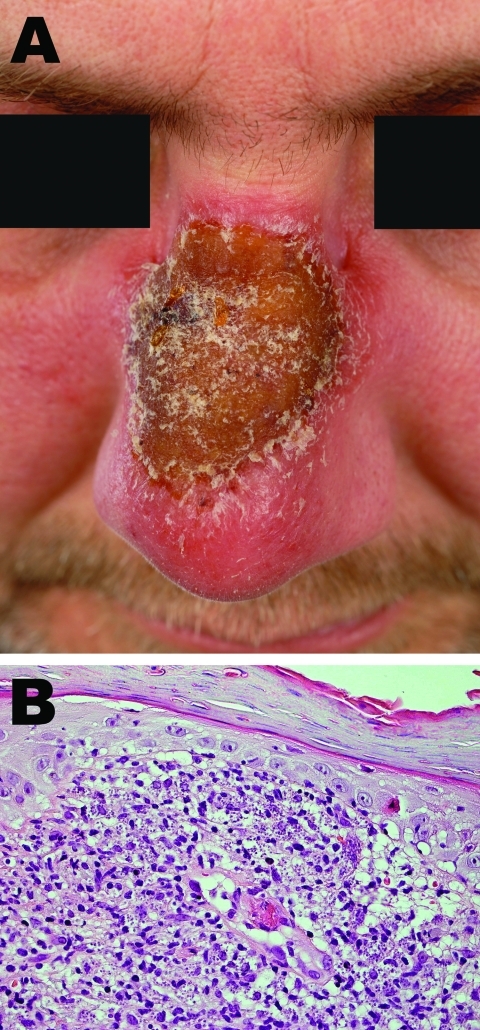
A) Crustosus plaque on the nose of the patient. B) The epidermis shows parakeratosis, atrophy, and a single apoptotic keratinocyte. An inflammatory infiltrate is present in the papillary dermis, mainly composed of (epithelioid) histiocytes, admixed with lymphocytes and few plasma cells. Most macrophages in the infiltrate are parasitized by numerous *Leishmania* spp. amastigotes (hematoxylin and eosin stain, original magnification ×400).

Revised histopathologic examination of the biopsy specimen showed a dermal inflammatory infiltrate of histiocytes containing numerous intracellular *Leishmania* amastigotes and epithelioid cells, lymphocytes, and few plasma cells (Figure, panel B). A direct smear from the biopsy specimen was positive for Leishman-Donovan bodies. Culture on Novy-MacNeal-Nicolle medium was positive for *Leishmania* spp. A PCR result for *Leishmania* performed on a biopsy specimen from the lesion was positive; sequence analysis showed DNA of *Leishmania donovani/infantum* complex.

Treatment was initiated with oral itraconazole (100 mg, 2×/d) for 6 weeks without improvement and was then continued with miltefosine (50 mg 3×/d) for 28 days. Other than nausea, the patient did not experience side effects. Regular monitoring of liver function showed values within normal limits. The lesion healed completely.

There are several reports of leishmaniasis acquired in Europe in locations north of the Mediterranean basin. Naucke et al. (*5*) reported 11 cases of endemically acquired leishmaniasis (human, canine, feline, and equine infections) in Germany since 1991. In 1992, a child with visceral leishmaniasis was described who had spent weekends and holidays near Calais, France (*6*).

We assume that our patient acquired cutaneous leishmaniasis in mainland Europe at 46°N. He had not visited Leishmaniasis-endemic areas before this holiday in the French Jura.

Cutaneous leishmaniasis in France is found mainly in the region Pyrénées-Orientales, with 2 sandflies, *Phlebotomus ariasi* and *Phlebotomus perniciosus*, as vectors (*7*). One of the causative factors for the northward emergence of leishmaniasis in Europe is the spread of visceral and cutaneous leishmaniasis from disease-endemic areas in the Mediterranean to neighboring temperate areas with vectors without disease (*8*). A northward spread of leishmaniasis with an extension of the geographic range of *Ph. perniciosus* and *Ph. neglectusus* sandflies has been found in Italy (*9*), and northward spread of the proven sanfly vector *Ph. (Laroussius) perniciosus* and the competent sandfly vector *Ph. (Transphlebotomus) mascittii* into Germany (*5*). It has been hypothesized that sandflies have always been sporadically present in central Europe, but that climate change will lead to extended distribution (*10*).

It is tempting to assume that climate change resulted in cutaneous leishmaniasis at 46°N in France. In any event, our case and those reported by others should make clinicians aware of the possibility of cutaneous leishmaniasis outside the well-known disease-endemic areas.
