# Correction: Vol. 18 No. 1

**DOI:** 10.3201/eid1805.C11805

**Published:** 2012-05

**Keywords:** Correction: Vol. 18 No. 1, Erratum, Faber, 11-0408

Author Henry J.C. de Vries’ initials were listed incorrectly in Cutaneous Leishmaniasis Acquired in Jura, France (W.R. Faber et al.). The article has been corrected online (wwwnc.cdc.gov/eid/article/18/1/11-0408_article.htm).

